# Comparative Studies on Chemical Contents and Effect in Kidney-Yang Deficiency Rats of Salt-Processed Product and Wine-Processed Product of Cuscutae Semen

**DOI:** 10.1155/2019/2049497

**Published:** 2019-08-29

**Authors:** Ying Zhang, Shu-ya Xu, Meng-nan Liu, Tian-ying Jia, Wen-jia Qu, Ting Han, Zhe Jia, Xin-fang Xu, Xiang-ri Li

**Affiliations:** ^1^School of Chinese Materia Medica, Beijing University of Chinese Medicine, Higher Education Garden, Liangxiang, Fangshan District, Beijing 102488, China; ^2^College of Pharmacy Engineering, Henan University of Animal Husbandry and Economy, Zhengzhou 450046, China

## Abstract

Cuscutae Semen mainly includes salt-processed product (SPP) and wine-processed product (WPP), which are most commonly used in traditional Chinese medicine. However, the differences between SPP and WPP have not been reported. In this paper, comparative studies between SPP and WPP on chemical contents and effect in kidney-yang deficiency rats have been investigated. UPLC-MS/MS was used to study the differences in chemical components. Kidney-yang deficiency was induced by hydrocortisone in rats. Rats were orally administrated with different doses of Jinkui Shenqi Pills, SPP, and WPP for 28 days. The average organ coefficients, renal function indexes, sex hormone levels, and testicular morphology were detected. As a result, the contents of flavonoids and chlorogenic acids were higher in SPP than in WPP, which may be caused by different processing methods. The improvement on reproduction of SPP was reflected in organ coefficients, renal function indexes, and biochemical properties of seminal plasma; furthermore, WPP was in sex hormone levels and morphology of testis. As a conclusion, these results indicated that both SPP and WPP can improve the reproductive function of kidney-yang deficiency rats with different mechanisms, which may be due to the differences in chemical contents between WPP and SPP as well as different processing methods. It is the first time that the differences between SPP and WPP have been studied in reproductive function in rats with kidney-yang deficiency.

## 1. Introduction

Cuscutae Semen (the dry mature seed of *Cuscuta australis* R. Br. or *Cuscuta chinensis* Lam., belonging to the family *Convolvulaceae*) is first recorded in the “*Shen Nong's Herbal*” as an upper-grade drug about 2000 years ago [1]. It is widely applied in Chinese medicine practice to nourish the liver and kidney, treat impotence and seminal emission, prevent miscarriage, and improve eyesight [2–4].

In order to ensure the safety and effect in clinical application, most of the herbal drugs are always processed (known as Pao Zhi in Chinese) before prescription [5]. It is believed that herbal drugs after processing can change the therapeutic range, enhance the effect, or reduce toxicity in traditional Chinese medicine (TCM). The processing of Cuscutae Semen, mainly including salt-processed product (SPP) and wine-processed product (WPP), has been applied for a long history [1, 6]. WPP is the initial processed product of Cuscutae Semen, which is first recorded in “*A Handbook of Formulas for Emergencies*” (Zhou Hou Bei Ji Fang) in the Jin Dynasty of China [7]. SPP is first documented in “*Comprehensive Recording of Sage-Like Benefit*” (Sheng Ji Zong Lu) in the North Song Dynasty [8]. There is different understanding on the effect of SPP and WPP by the theory of TCM. The salt processing is supposed to lead drugs into channel tropism of kidney and enhance functions of the kidney, such as nourishing kidney and improving reproductive function. Wine processing is presumed to change the property of medicine and promote yang-qi, such as kidney-yang [5]. SPP is more adept at nourishing kidney and astringent while WPP can enhance the role of warming spleen and kidney [5]. However, to the best of our knowledge, no study on differences between SPP and WPP has been described. Based on the works we had done before, this paper compared chemical compositions and reproductive function in rats with kidney-yang deficiency between SPP and WPP for the first time. We expect to find the theoretical basis for the processing of traditional Chinese medicine.

Previous phytochemical investigations on Cuscutae Semen have led to the isolation of a series of natural products, including flavonoids, chlorogenic acids, and polysaccharides [9, 10]. The flavonoids and chlorogenic acids are reported to be biologically active constituents in Cuscutae Semen [1, 3, 11]. Cuscutae Semen flavonoids can improve reproductive injury induced by glycosides of *Tripterygium wilfordii* Hook.f. [9], regulate endocrine dysfunction (sex hormone receptors) [12], ameliorate kidney-yang deficiency symptoms [13], and improve the immune function of kidney-yang deficiency rats [14]. Modern pharmacological experiments have indicated that chlorogenic acids have antiviral effects and protective effects on the liver injury and gallbladder [11]. Our previous study reveals that the contents and biological activities of total flavonoids between the crude products (CP) and SPP of Cuscutae Semen are different [1]. Flavonoids and chlorogenic acids are also identified by UPLCs in our previous study [15]. However, up to now, the comparative studies on chemical contents and effect between SPP and WPP have not been described. In this study, the contents of bioactive chemicals are determined to differentiate chemical components between SPP and WPP.

As the symptom of kidney-yang deficiency rats is similar to the kidney-yang deficiency in TCM, hydrocortisone-induced kidney-yang deficiency rats are used for comparative studies on reproductive function between SPP and WPP [16].

## 2. Materials and Methods

### 2.1. Chemicals and Reagents

**Figure 1:**
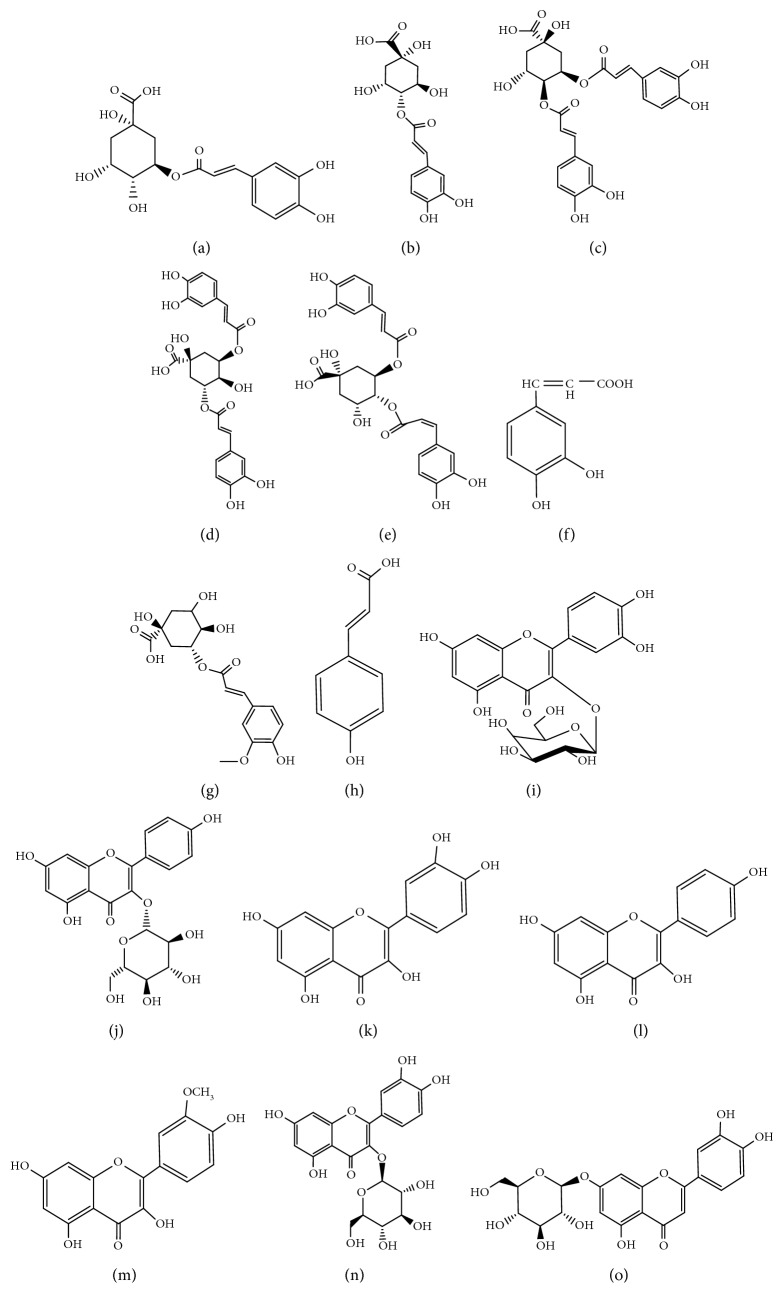
Chemical formula of flavonoids in Cuscutae Semen, (a) 3-O-caffeoylquinic acid, (b) 4-O-caffeoylquinic acid, (c) 3,4-di-O-caffeoylquinic acid, (d) 3,5-di-O-caffeoylquinic acid, (e) 4,5-di-O-caffeoylquinic acid, (f) caffeic acid, (g) 5-O-feruloylquinic acid, (h) *p*-hydroxycinnamic acid, (i) hyperoside, (j) astragalin, (k) quercetin, (l) kaempferol, (m) isorhamnetin, (n) isoquercitrin, and (o) luteolin-7-O-glucoside.

Hyperoside (≥98.0%), quercetin (≥98.0%), astragalin (≥98.0%), kaempferol (≥98.0%), and isorhamnetin (≥98.0%) were supplied from National Institutes for Food and Drug Control (Beijing, China). Luteolin-7-O-glucoside (≥98.0%), isoquercitrin (≥98.0%), 4-caffeoylquinic acid (4-CQA) (≥98.0%), 3-caffeoylquinic acid (3-CQA) (≥98.0%), 4,5-dicaffeoylquinic acid (4,5-DiCQA) (≥98.0%), 3,4-dicaffeoylquinic acid (3,4-DiCQA) (≥98.0%), 3,5-dicaffeoylquinic acid (3,5-DiCQA) (≥98.0%), caffeic acid (CA) (≥98.0%), *p*-hydroxycinnamic acid (≥98.0%), and 5-O-feruloylquinic acid (5-FQA) (≥98.0%) were purchased from Shanghai Yuanye Bio-Technology Co., Ltd. (Shanghai, China). Their structures (shown in Figure 1) were fully explicated by spectra data (ESI-MS), which followed the methods of Zhang et al. [15].

Acetonitrile (MS grade) and formic acid (MS grade) were supplied from Thermo Fisher Scientific Inc. All the other chemicals of analytical grade were commercially available from Beijing Chemical Works (Beijing, China). Other reagents were of analytical grade. Deionized water for preparing samples was purified by MilliQ50 SP Reagent Water System (Bedford, MA, USA). Distillated water for mobile phase was purchased from A. S. Watson Group (Hong Kong) Ltd.

### 2.2. Collection of Samples

We followed the methods of Zhang et al. [15]. The crude products of Cuscutae Semen (lot number: 160161211) were obtained from Beijing Kangmei Pharmaceutical Co., Ltd. (Beijing, China) and authenticated as Cuscutae Semen by Yang Yaojun, the professor of Pharmacognosy Department in Beijing University of Chinese Medicine. Voucher specimens were deposited in the School of Chinese Materia, Beijing University of Chinese Medicine. SPP (lot number: 160715) was prepared in the laboratory, which meant that the CP was mixed with salt solution and then stir-heated at 80°C in a metallic pan and dried in the air (2 kg salt per 100 kg of Cuscutae Semen). WPP (Lot number: 160716) samples were mixed with wine and then stir-heated at 100°C and dried in the air (20 kg wine per 100 kg of Cuscutae Semen).

### 2.3. Preparation of Samples

SPP and WPP were selected from the samples we collected and prepared in our lab (as described in Section 2.2). The samples were accurately weighed as 0.8 kg and soaked 8 times in 60% ethanol for one night. And then, they were extracted under reflux twice for 1 h each time. The filtrate was combined, and the ethanol was removed. The ethanol extracts were dried in vacuum at 60°C. Then, the ethanol extracts were obtained. The w/w extraction yield of SPP and WPP was 9.16% and 9.10%, respectively. A part of the ethanol extracts of SPP were used for content determination. Another part of the extracts were diluted in distilled water when the rats were treated orally. Sample preparation in WPP was the same.

### 2.4. Determination of Flavonoids and Chlorogenic Acids in SPP and WPP

#### 2.4.1. Sample Preparation

The extracts of SPP and WPP were diluted with methanol. All solvents were filtered through 0.22 *μ*m membranes prior to injection.

#### 2.4.2. Standards Preparation

Stock solutions were achieved by eight standards dissolved in methanol solvent, respectively. Then, the stock solutions were diluted into a concentration range of working solutions with methanol. The mean peak areas versus the concentrations of each compound were plotted to construct the calibration curves.

#### 2.4.3. Method Validation

- *Limit of Detection (LOD) and Limit of Quantification (LOQ)*. By injecting a series of dilutions of standards, LOD and LOQ were determined. LOD was considered as the peak area three times greater than the noise level. LOQ was the peak area ten times greater than the noise level.
- *Precision*. The precision was evaluated by six continuous injections of extracts of Cuscutae Semen within one day.
- *Repeatability*. Repeatability was evaluated by six parallel extracts of Cuscutae Semen.
- *Stability*. Stability of the investigated compounds was evaluated by analyzing the same sample at 0 h, 2 h, 6 h, 8 h, 12 h, and 24 h after being prepared.
- *Recovery*. Recovery was determined with an equal amount of the reference compounds adding to 0.5 g of Cuscutae Semen. Recovery (*R*%) was calculated according to the following equation: *R*% = (C1 − C2)/C3 × 100, where C1 was the concentration of actual measurement, C2 was the theoretical concentration, and C3 was the concentration of standard which was added.

#### 2.4.4. UPLC-MS/MS Method

The extracts were chromatographically separated on an Agilent Eclipse Plus C_18_ column (2.1 × 100 mm i.d., 1.8 *μ*m). The mobile phase consisted of A (acetonitrile) and B (water containing 0.05% formic acid, v/v). The flow rate was 0.30 mL/min. The elution conditions for flavonoid analysis were applied with a linear gradient as follows: 0–3 min, 12–12% A; 3–5 min, 12–15% A; 5–10 min, 15–15% A; 10–15 min, 15–40% A; and 15–18 min, 40–40% A. The column temperature was at room temperature.

The elution conditions for chlorogenic acid analysis were applied with a linear gradient as follows: 0–3 min, 6–6% A; 3–6 min, 6–18% A; 6–8 min, 18–22% A; 8–11 min, 22–27% A. The column temperature was at room temperature.

Detection was performed on an Agilent 1290 Agilent 6460 Triple Quad LC/MS (Agilent, American) with an ESI source. Targeted biomarkers were further separated and quantified in multiple reaction monitoring (MRM) with negative ionization mode. The optimized MS conditions were as follows: gas temperature: 300°C, gas flow: 7 L/min, nebulizer: 35 psi, sheath gas temperature: 250°C, sheath gas flow: 11 L/min, capillary volt: 3500 V, nozzle volt: 500 V, and chamber current: 0.19 *μ*A.

The transitions for flavonoids were *m/z* 463.1 → 301.0 (hyperoside), *m*/*z* 447.1 ⟶ 255.0 (astragalin), *m*/*z* 301.04 ⟶ 151.0 (quercetin), *m*/*z* 285.04 ⟶ 93.0 (kaempferol), *m*/*z* 315.05 ⟶ 300.0 (isorhamnetin), *m*/*z* 463.1 ⟶ 301.0 (isoquercitrin), and 447.1 ⟶ 285.0 (luteolin-7-O-glucoside), respectively. The optimized collision energy for hyperoside, astragalin, quercetin, kaempferol, isorhamnetin, isoquercitrin, and luteolin-7-O-glucoside was 25, 30, 20, 40, 25, 20, and 25 eV, respectively. The retention time between astragalin and luteolin-7-O-glucoside was different, which meant that luteolin-7-O-glucoside flowed preferentially at C_18_ RP column. Also, hyperoside flowed more preferentially than isoquercitrin at C_18_ RP column.

The transitions for chlorogenic acids were *m*/*z* 353.0 ⟶ 190.1 (3-CQA), *m*/*z* 353.0 ⟶ 172.9 (4-CQA), *m*/*z* 515.1 ⟶ 173.0 (3,4-DiCQA), *m*/*z* 515.1 ⟶ 353.0 (3,5-DiCQA), *m*/*z* 515.1 ⟶ 353.0 (4,5-DiCQA), *m*/*z* 163.0 ⟶ 119.0 (*p*-hydroxycinnamic acid), 179.0 ⟶ 134.9 (CA), and 193.0 ⟶ 133.8 (5-FQA), respectively. The optimized collision energy for 3-CQA, 4-CQA, 3,4-DiCQA, 3,5-DiCQA, 4,5-DiCQA, *p*-hydroxycinnamic acid, CA, and 5-FQA was 10, 10, 25, 20, 15, 25, 15, and 15 eV, respectively. The retention time between 3-CQA and 4-CQA were different, which meant that 3-CQA flowed preferentially at C_18_ RP column. Also, 3,5-DiCQA flowed more preferentially than 4,5-DiCQA at C_18_ RP column.

### 2.5. Effect of Reversing the Kidney-Yang Deficiency

#### 2.5.1. Animals and Housing

Male Wistar rats (160–180 g) were obtained from SPF (Beijing) Biotechnology Co., Ltd. Before the experiment, all rats were allowed to acclimatize in environmentally controlled quarters (temperature of 21–23°C, relative humidity of 45–65%, and 12:12 h light/dark cycle) with food and water freely available for one week. The experiment complied with the ethical guidelines suggested by the Institutional Animal Ethics Committee and Committee for the Purpose of Control and Supervision of Experiments on Animals, Ministry of Health, and Government of China.

#### 2.5.2. Treatment of Animals

Sixty rats were randomly partitioned into five groups. Four groups were given an intramuscular injection of 20 mg/kg hydrocortisone sodium succinate (purchased from Tianjin Biochem Pharmaceutical Co., Ltd., Tianjin, China) for 14 days to establish the kidney-yang deficiency model except for normal control group (NC) which was injected with an equal volume of physiological saline. After this treatment, the kidney-yang deficiency model was made.

On the 15th day, five groups were treated as follows: Jinkui Shenqi Pills group (JK) was treated orally at dose of 105 mg/kg/d Jinkui Shenqi Pills (purchased from Beijing Tongrentang Group Co., Ltd., Beijing, China); SPP group was treated orally with 0.18 g/kg ethanol extracts of SPP; WPP group was treated orally with 0.18 g/kg ethanol extracts of WPP; model group (M) and normal control group (NC) were treated with an equal volume of distilled water. All the samples were administered by gastric perfusion.

Twenty-four hours after the last treatment (after 4 weeks' treatment), all rats were anesthetized and sacrificed. Blood samples were collected from abdominal aorta into Eppendorf tubes and then were centrifuged at 3000 rpm for 15 min to collect the serum and then stored at −80°C until the determination of renal function indexes and hormone levels. One kidney and one testicle were rapidly removed and weighed as well as epididymis, prostate gland, and seminal vesicle. The rest of the testicles were fixed in Bouin's fixative solution for 24 h at 4°C, which were subsequently embedded in paraffin using standard procedures and stained with hematoxylin-eosin (HE).

#### 2.5.3. Weight Analysis

During the testing, the rats were weighed once a week for adjusting the dosage and weighed on the last day.

#### 2.5.4. Analysis of Renal Function Indexes

Before the rats were executed, their serums were collected. Serum creatinine (CRE), blood urea nitrogen (BUN), and uric acid (UA) levels were determined in the Scientific Research Center of Beijing University of Chinese Medicine.

#### 2.5.5. Hormone Analyses

Total serum testosterone (T), estradiol (E_2_), luteinizing hormone (LH), and follicle-stimulating hormone (FSH) were determined by radioimmunoassay using the Testosterone radioimmunoassay kit and Estradiol radioimmunoassay kit (Nanjing Jiancheng Bioengineering Institute), Rat LH ELISA Kit, and Rat FSH ELISA Kit following the instructions (CUSABIO Engineering Co., LTD).

#### 2.5.6. Determination of Biochemical Properties of Seminal Plasma

Seminal vesicle fluid fructose, acid phosphatase, *α*-glucosides, and seminal plasma zinc were determined by radioimmunoassay using the Fructose kit (Nanjing Jiancheng Bioengineering Institute), Rat ACP ELISA kit (Nan Jing Sen Bei Jia Biotechnology Co., LTD.), Rat *α*-Glu ELISA kit (Nan Jing Sen Bei Jia Biotechnology Co., LTD.), and Zinc assay kit (Nan Jing Sen Bei Jia Biotechnology Co., LTD.) following the instructions.

#### 2.5.7. Morphological Observation of Testis

The left testicles were washed twice with phosphate-buffered saline (PBS). After fixed 1 h in Bouin's fixative, they were stained with hematoxylin-eosin (HE), followed by examination for histopathological changes under the microscope.

### 2.6. Statistical Analysis

The UPLC-MS/MS data for quantitative analysis were acquired and processed using the Agilent Mass Hunter Workstation Software (version B.07.00).

Data for animal experiments were expressed as mean ± standard deviations and analyzed with one-way analysis of variance (ANOVA) by SAS software 9.3. *P* < 0.05 was considered statistically significant.

## 3. Results

### 3.1. Quantitative Analysis by UPLC-MS/MS

#### 3.1.1. Method Validation

The regression equations, together with LOD and LOQ values for the quantitation method, are shown in Table 1. The calibration curves were in good linearity with correlation coefficients over 0.9900. The RSD for peak area was within 5.79%, which suggested that the investigated compounds were stable within 24 h. The RSD of repeatability and precision was within 3.86% and 1.96% (Table 1), respectively, which suggested that the method was reproducible and precise. The recovery test (Table 2; recovery ranged from 101.69% to 104.80%) suggested good accuracy of the method. The results were validated by the reliability of the method.

#### 3.1.2. Contents Analysis in SPP and WPP

The contents of fifteen major compounds in SPP and WPP of Cuscutae Semen samples are shown in Table 3. The results showed a significant difference between the two processed products. Except for quercetin, SPP had a higher content of other 14 components than WPP. 3-CQA and hyperoside were considered as the major constituents in SPP and WPP.

### 3.2. Effect of Reversing the Kidney-Yang Deficiency

#### 3.2.1. Kidney-Yang Deficiency Model Was Successfully Made

After intramuscular injection of hydrocortisone sodium succinate for 14 days, the rats showed withered clothing hair, decreases in body weight, slowed reaction, aversion cold, weakness, tendency to cluster, and decreased activity as in kidney-yang deficiency. Based on these clinical parameters, the model was regarded as successful.

#### 3.2.2. Average Organ Coefficients

**Figure 2:**
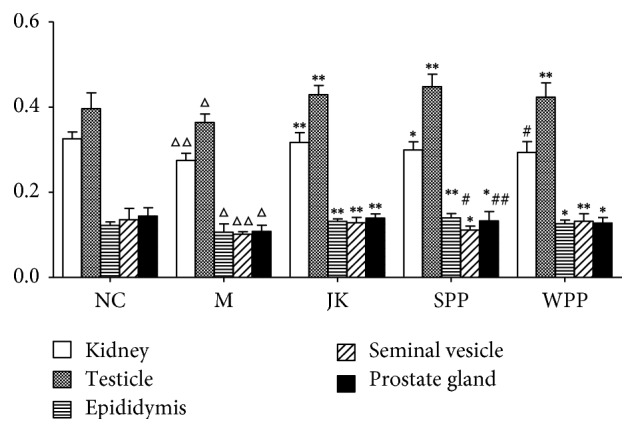
Average organ coefficients (100%) in each group. Significant differences with the NC group were designated as ^Δ^*P* < 0.05, ^ΔΔ^*P* < 0.01, with the M group ^*∗*^*P* < 0.05, ^*∗∗*^*P* < 0.01, with the WPP group ^#^*P* < 0.05, ^##^*P* < 0.01.

All rats and their organ were weighed, and the average organ coefficients were calculated. Compared with the normal control group, the coefficients of kidney, testicle, epididymis, seminal vesicle, and prostate gland were decreased in the model group (*P* < 0.05 or *P* < 0.01). Compared with the model group, the organ coefficients of the JK, SPP, and WPP groups were increased. The SPP group was better in the epididymis and prostate gland than the WPP group (*P* < 0.05 or *P* < 0.01). The results are shown in Figure 2 and Table 4.

#### 3.2.3. Analysis of Renal Function Indexes

**Figure 3:**
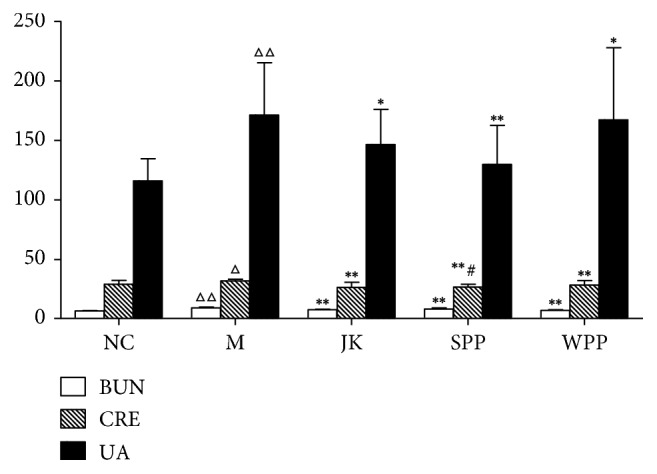
Effect of each group on renal function. Significant differences with the NC group were designated as ^Δ^*P* < 0.05, ^ΔΔ^*P* < 0.01, with the M group ^*∗*^*P* < 0.05, ^*∗∗*^*P* < 0.01, with the WPP group ^#^*P* < 0.05.

In traditional Chinese medicine, the kidney is thought to be related to reproduction. Based on the theory, reproductive ability, growth, and development were determined by the essence and vitality of the kidney [17]. The detection of renal function can be used to evaluate the reproductive function in kidney-yang deficiency rats. As shown in Table 5 and Figure 3, the levels of CRE, BUN, and UA in the serum were determined. Compared with the NC group, the levels of CRE, BUN, and UA were increased in the model group (*P* < 0.05 or *P* < 0.01). Compared with the M group, the CRE, BUN, and UA levels of all treated groups were lower (*P* < 0.05 or *P* < 0.01). The contents of CRE were significantly different between SPP and WPP. The SPP group had better renal injury improvement than the WPP group.

#### 3.2.4. Hormone Analyses

**Figure 4:**
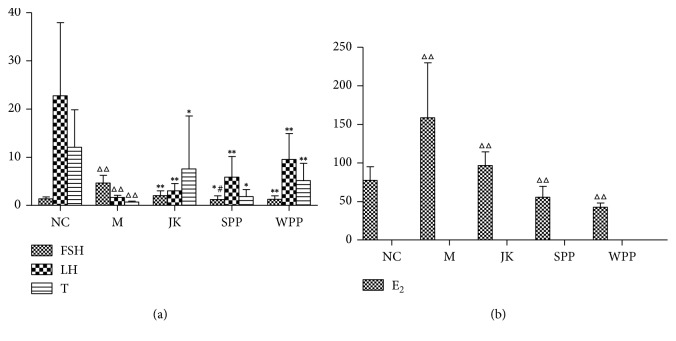
Sex hormone levels in each group. Significant differences with the NC group were designated as ^ΔΔ^*P* < 0.01, with the M group ^*∗*^*P* < 0.05, ^*∗∗*^*P* < 0.01, with the WPP group ^#^*P* < 0.05.

Compared with the normal control group, testosterone (T) and luteinizing hormone (LH) levels in the serum of the model group were lower. Estradiol (E_2_) and follicle-stimulating hormone (FSH) levels were higher in the model group (*P* < 0.01). Compared with the M group, T and LH levels increased while E_2_ and FSH levels decreased in the SPP and WPP groups as shown in Figure 4 and Table 6.

#### 3.2.5. Analysis of Biochemical Properties of Seminal Plasma

**Figure 5:**
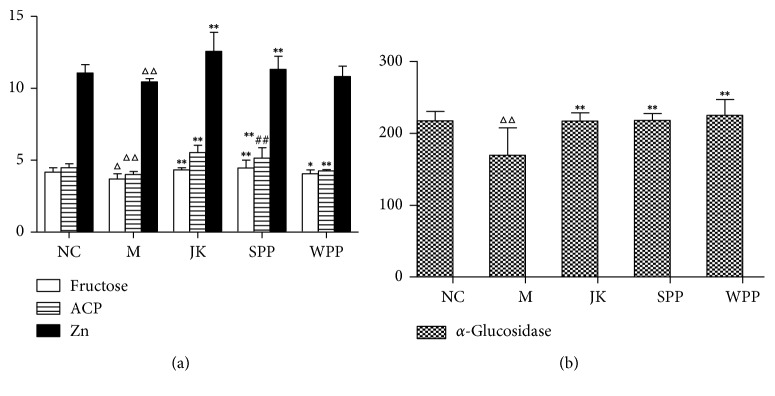
Biochemical indexes of seminal plasma in rats. Significant differences with the NC group were designated as ^Δ^*P* < 0.05, ^ΔΔ^*P* < 0.01, with M group ^*∗*^*P* < 0.05, ^*∗∗*^*P* < 0.01, with WPP group ^#^*P* < 0.05, ^##^*P* < 0.01.

As shown in Figure 5 and Table 7, compared with the NC group, the levels of fructose, acid phosphatase (ACP), *α*-glucosidase, and zinc in the seminal vesicle of the model group were decreased (*P* < 0.05 or *P* < 0.01). Compared with the M group, biochemical indexes of seminal plasma in all treated groups were increased (*P* < 0.05 or *P* < 0.01). The contents of fructose, ACP, and Zn were significantly increased in the SPP group compared with the WPP group. The SPP group had improved this condition better than the WPP group.

#### 3.2.6. Morphological Observation of Testis

**Figure 6:**
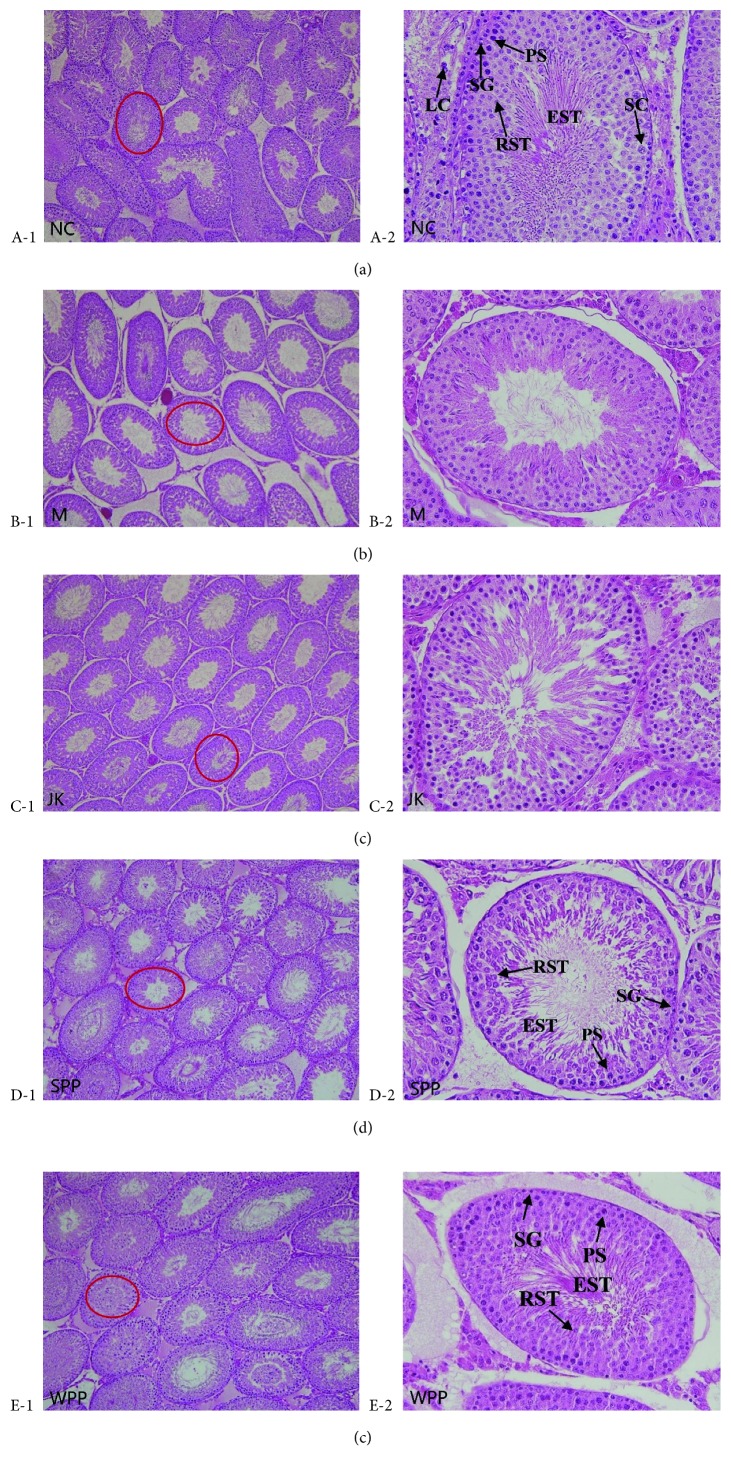
Histological observation of testis from experimental rats was examined using H&E staining and microscopy (100x and 400x magnification). Leydig cell (LC); Sertoli cell (SC); spermatogonia (SG); primary spermatocytes (PS); round spermatids (RST); elongated spermatids (EST). Graphs marked above by circle are enlarged right and marked as A2-E2. NC (A-1, 100x; A-2, 400x); M (B-1, 100x; B-2, 400x); JK (C-1, 100x; C-2, 400x); SPP (D-1, 100x; D-2, 400x); WPP (E-1, 100x; E-2, 400x).

Testicular histology was regular in the NC group (Figure 6 A-1, A-2). There was no evidence of disruption or injury among the seminiferous tubules. The seminiferous tubules were arranged compactly. The spermatogenic cells were well developed. The spermatogonia (SG), primary spermatocytes (PS), round spermatids (RTS), and elongated spermatids (EST) were observed. The number of cells was at normal levels and the cell layer was clear. There were visible mature sperms within the lumen, rich mesenchymal cells, and clear and complete structures.

Of the model group (Figure 6 B-1, B-2), the seminiferous cells were significantly lower and the cells were loose. The spermatogenic cells were arranged disorderly and were found shortened. Cell nuclei were seriously sparse and vacuoles were existed. The sperm counts had significantly dropped. The stromal cells were atrophy with the number decreased. Based on these pathologies, the kidney-yang model was successfully made.

In the JK group (Figure 6 C-1, C-2), compared with the model group, the seminiferous tubules were arranged more compactly. The number of spermatogenic cells increased. The cells were arranged in a neat and orderly manner. Leydig cells were abundant; meanwhile, the degeneration degree was improved.

In the SPP group (Figure 6 2 D-1, D-2), the seminiferous tubules were arranged compactly. Compared with the model group, the number of spermatogenic cells increased and the cells were arranged in order. With improved degradation degree and increased number of Leydig cells, vacuoles were still existed.

In the WPP group (Figure 6 E-1, E-2), compared to the model group, the seminiferous tubules were arranged compactly and the number of spermatogenic cells increased. There were visible mature sperms within the lumen. With improved degradation degree and a rich number of Leydig cells, the vacuoles were improved obviously.

## 4. Discussion

Based on the TCM theory, kidney-yang is essential for human growth and development as well as reproductive function. The kidney-yang deficiency syndromes are used in TCM to elucidate a status in which the patients show a group of symptoms mainly including soreness and weakness of waist and knees, fatigue, cold feeling of the whole body, impairment of hearing, looseness of teeth, and even reproductive dysfunction [14]. In this study, hydrocortisone-induced kidney-yang deficiency rats were used for a comparative study on reproductive function between SPP and WPP, in which rats will show similar symptoms with those have been described in TCM kidney-yang deficiency [16]. The hydrocortisone-induced kidney-yang deficiency symptoms rats are commonly used to evaluate the pharmacological activity of reinforcing kidney-yang herbs [18].

Cuscutae Semen is a traditional Chinese medicine commonly used in reinforcing kidney-yang and alleviating or even reversing the symptoms of kidney-yang deficiency. The processing of Cuscutae Semen, mainly including salt-processed product (SPP) and wine-processed product (WPP), has been applied for a long history. However, no study has investigated the differences between SPP and WPP. Our previous study showed that phenolic compounds are the major constituents of Cuscutae Semen. Flavonoids and chlorogenic acids were identified [15]. In this study, we determined fifteen major compounds in SPP and WPP including seven flavonoids and eight chlorogenic acids. Compared with WPP, most of the contents of fifteen major compounds were higher in SPP. The content distinction may be caused by different processing methods. We speculate that the content differences of chemical composition between SPP and WPP may be related to the effect differences.

The value of organ coefficients is equal to the percentage of the body weight divided by the absolute organ weight. In this study, the organ coefficients of the SPP and WPP groups were increased, which indicated that SPP and WPP could reverse organ coefficients decline. In general, the effect of SPP was better than that of WPP.

Clinically, serum CRE, BUN, and UA are important indexes to evaluate renal function [19, 20]. When the renal function is disrupted, CRE, BUN, and UA increase. In this study, the SPP and WPP groups could decrease the contents of CRE, BUN, and UA. It indicated that the processed products of Cuscutae Semen were able to improve the renal injury induced by hydrocortisone. The experimental results showed that the SPP group mainly reduced the CRE content. Overall, SPP is better to alleviate kidney damage than WPP.

Experimental studies [21] indicate that kidneys are closely correlated with the reproductive endocrine system. FSH, LH, T, and E_2_ are important indexes to evaluate the reproductive function. The results showed that hydrocortisone caused gonadal and reproductive function injury. SPP and WPP regulated the levels of sex hormones (increase E_2_ and FSH and decrease T and LH) and improved testis injury in kidney-yang deficiency rats, so as to regulate the disturbance of reproductive function. In general, the effect of WPP on the regulation of sex hormone levels was stronger than that of SPP.

Seminal vesicle which plays an important role in male reproduction is an important accessory gland in the male reproductive system. With the development of biochemical technology, the evaluation of seminal plasma biochemical indexes and the function of male accessory gonad on male reproductive ability have been paid more and more attention. *α*-Glucosidase, ACP, fructose, and Zn are major indexes to evaluate the function of male reproductive ability [22–24]. The results showed that processed products of Cuscutae Semen improved the reproductive function in rats by enhancing the accessory gonad function of kidney-yang deficiency rats. SPP was more adept at enhancing seminal plasma biochemical indexes than WPP.

The testis is the major organ of the male reproductive system. Experimental studies have shown that the kidney-yang deficiency can affect the morphology of testicular tissue [25]. In the model group, the seminiferous tubules showed obvious damage, indicating that the model of kidney-yang deficiency was successfully established. The testes of the Cuscutae Semen processed products group rats were improved. Based on the results, the effect of the WPP group was better than that of the SPP group.

Total flavonoids, efficacious components of Cuscutae Semen, have been reported to show regulatory effects on reproductive and endocrine function [12, 26], such as regulating LH, T, and E_2_ levels. Chlorogenic acids, other active components, exhibited protective effects on sperm plasma [27, 28]. Previous studies have also shown that quercetin, astragalin, kaempferol, feruloylquinic acid, and caffeic acid have beneficial and/or protective effects on improving the reproductive function, preventing sperm damage, ameliorating sperm function, and improving testicular function [27–32]. In this study, SPP and WPP showed protective effects on reproductive, endocrine, and testicular function. The protective effects may be related to these components which need further investigation.

## 5. Conclusions

The contents of flavonoids and chlorogenic acids were higher in SPP than in WPP. The content distinction may be caused by different processing methods. Pharmacological experimental results demonstrated that both SPP and WPP could improve the reproductive function in kidney-yang deficiency rats. The improvement on reproduction of SPP was reflected in organ coefficients, renal function indexes, and biochemical properties of seminal plasma; furthermore, WPP was in sex hormone levels and morphology of testis. The different improvement mechanism may be due to the differences in chemical contents between WPP and SPP as well as different processing methods. The pharmacological experiment results are in accordance with the theory of Cuscutae Semen processing, which provides the theoretical basis for the clinical application of Cuscutae Semen and the further development and utilization. However, the action mechanism of the salt-processed product and wine-processed product of Cuscutae Semen needs further study.

## Figures and Tables

**Table 1:** Method validation of fifteen analytes in UPLC-MS quantitation.

Number	*t*_R_ (min)	Analytes	Formula	Regression equation	*r* ^2^	Linear range (ng/ml)	LOD (*μ*g/mL)	LOQ (*μ*g/mL)	Precision (*n* = 6)	Repeatability (*n* = 6)	Stability (0–24 h)
1	5.51	3-CQA	C_16_H_18_O_9_	*y* = 0.0775*x* − 2872.8378	0.9988	20341–244093	0.0014	0.0041	1.96	3.86	5.79
2	16.73	4-CQA	C_16_H_18_O_9_	*y* = 0.1345*x* − 4699.9042	0.9991	3872–46461	0.0028	0.0084	0.33	0.44	0.57
3	1.46	3,4-DiCQA	C_25_H_23_O_12_	*y* = 0.3181*x* − 65.9357	0.9990	206–2478	0.0112	0.0225	0.0019	0.0017	0.02
4	2.34	3,5-DiCQA	C_25_H_23_O_12_	*y* = 0.2140*x* − 56.4874	0.9997	462–7394	0.4436	1.3309	0.0037	0.013	0.06
5	2.93	4,5-DiCQA	C_25_H_23_O_12_	*y* = 0.1230*x* − 274.8306	0.9974	477–7636	0.4582	1.3745	0. 01	0.03	0.31
6	5.85	CA	C_9_H_8_O_4_	*y* = 0.0003*x* − 0.4863	0.9915	5–75	0.0026	0.0105	0.03	0.07	0.16
7	7.73	5-FQA	C_10_H_10_O_4_	*y* = 0.3482*x* − 18.5959	0.9998	206–2470	0.0001	0.0004	0.0019	0.0006	0.0072
8	4.04	*p*-Hydroxycinnamic acid	C_9_H_8_O_3_	*y* = 0.1221*x* − 345.2182	0.9921	488–7806	0.0004	0.0014	0.03	0.03	0.37
9	10.03	Hyperoside	C_21_H_20_O_12_	*y* = 0.0638*x* − 4194.4234	0.9991	6833–109333	0.0036	0.0109	—	—	—
10	13.29	Astragalin	C_21_H_20_O_11_	*y* = 0.1698*x* − 10717.3813	0.9995	2683–42933	0.0172	0.0687	0.13	0. 4	0.09
11	23.75	Quercetin	C_15_H_10_O_7_	*y* = 0.0035*x* − 69.1497	0.9989	202–2424	0.0006	0.0012	0.28	0.43	0.64
12	27.77	Kaempferol	C_15_H_10_O_6_	*y* = 0.2544*x* − 406.6164	0.9974	5684–68210	0.0008	0.0017	0.24	0.09	0.63
13	28.54	Isorhamnetin	C_16_H_12_O_7_	*y* = 0.0190*x* − 1032.1204	0.9967	1351–16210	8.76*E* − 05	0.0004	0.32	0.73	1.18
14	10.4	Isoquercitrin	C_21_H_20_O_12_	*y* = 0.0473*x* − 2285.4629	0.9990	2567–41067	0.0020	0.0082	1.26	0.50	1.29
15	16.12	Luteolin-7-*O*-glucoside	C_21_H_20_O_11_	*y* = 0.4190*x* − 2399.9345	0.9978	2196–26358	0.0004	0.0013	0.06	0.02	0.02

In the regression equation, *x* is the peak area, *y* the concentration of each analyte (ng/mL), and *r* the correlation coefficient. LOD, limit of detection (S/N = 3); LOQ, limit of quantification (S/N = 10). Precision, repeatability, and stability are shown in RSD (%).

**Table 2:** Recovery of the analytes (*n* = 6).

Number	*t*_R_ (min)	Analytes (%)	Initial amount (mg)	Added amount (mg)	Detected amount (mg)	Recovery (%)	RSD
1	5.51	3-CQA	4.9141	5.0000	10.0126	1.0169	0.0124
2	16.73	4-CQA	0.8283	0.8195	1.6676	1.0242	0.0158
3	1.46	3,4-DiCQA	0.1043	0.1033	0.2103	1.0263	0.0192
4	2.34	3,5-DiCQA	0.0539	0.0529	0.1084	1.0345	0.0166
5	2.93	4,5-DiCQA	0.0672	0.0665	0.1360	1.0345	0.0077
6	5.85	CA	0.0320	0.0341	0.0667	1.0173	0.0197
7	7.73	5-FQA	0.0089	0.0089	0.0181	1.0277	0.0127
8	4.04	*p*-Hydroxycinnamic acid	0.0775	0.0766	0.1557	1.0209	0.0113
9	10.03	Hyperoside	1.3739	0.7216	2.1176	1.0306	0.0116
10	13.29	Astragalin	0.2940	0.2962	0.5958	1.0185	0.0108
11	23.75	Quercetin	0.1188	0.1183	0.2410	1.0332	0.0174
12	27.77	Kaempferol	0.7358	0.2592	1.0044	1.0362	0.0052
13	28.54	Isorhamnetin	0.0645	0.0642	0.1317	1.0480	0.0031
14	10.4	Isoquercitrin	0.3267	0.3203	0.6571	1.0316	0.0081
15	16.12	Luteolin-7-*O*-glucoside	0.1441	0.1377	0.2862	1.0318	0.0094

**Table 3:** The contents of fifteen major compounds in SPP and WPP of Cuscutae Semen (mg/g, mean ± SD, *n* = 2).

Number	*t*_R_ (min)	Analytes	SPP (mg/g)	WPP (mg/g)
1	5.51	3-CQA	9.8436 ± 0.3004	8.9619 ± 0.1361
2	16.73	4-CQA	1.8759 ± 0.0525	1.1195 ± 0.0335
3	1.46	3,4-DiCQA	0.0269 ± 0.0008	0.0167 ± 0.0004
4	2.34	3,5-DiCQA	0.1418 ± 0.0029	0.0772 ± 0.0022
5	2.93	4,5-DiCQA	0.2977 ± 0.0047	0.1463 ± 0.0039
6	5.85	CA	0.0877 ± 0.0018	0.0448 ± 0.0011
7	7.73	5-FQA	0.0176 ± 0.0007	0.0159 ± 0.0007
8	4.04	*p*-Hydroxycinnamic acid	0.1999 ± 0.0022	0.1403 ± 0.0040
9	10.03	Hyperoside	2.9477 ± 0.0358	2.5337 ± 0.0215
10	13.29	Astragalin	0.6622 ± 0.0102	0.4586 ± 0.0102
11	23.75	Quercetin	0.3196 ± 0.0047	0.3806 ± 0.0112
12	27.77	Kaempferol	1.8872 ± 0.0447	1.3468 ± 0.0360
13	28.54	Isorhamnetin	0.1656 ± 0.0034	0.1197 ± 0.0032
14	10.4	Isoquercitrin	0.6841 ± 0.0083	0.6074 ± 0.0144
15	16.12	Luteolin-7-*O*-glucoside	0.3091 ± 0.0072	0.2673 ± 0.0093

**Table 4:** The average organ coefficients (100%).

	Kidney	Testicle	Epididymis	Seminal vesicle	Prostate gland
NC	0.3256 ± 0.0160	0.3961 ± 0.0373	0.1223 ± 0.0078	0.1356 ± 0.0262	0.1443 ± 0.0190
M	0.2745 ± 0.0171^ΔΔ^	0.3638 ± 0.0198^Δ^	0.1061 ± 0.0198^Δ^	0.1013 ± 0.0062^ΔΔ^	0.1081 ± 0.0144^Δ^
JK	0.3170 ± 0.0227^*∗∗*^	0.4290 ± 0.0216^*∗∗*^	0.1315 ± 0.0061^*∗∗*^	0.1284 ± 0.0127^*∗∗*^	0.1395 ± 0.0098^*∗∗*^
SPP	0.2991 ± 0.0193^*∗*^	0.4478 ± 0.0294^*∗∗*^	0.1397 ± 0.0103^*∗∗#*^	0.1111 ± 0.0096^*∗*^	0.1327 ± 0.0220^*∗##*^
WPP	0.2935 ± 0.0253^*#*^	0.4234 ± 0.0334^*∗∗*^	0.1262 ± 0.0085^*∗*^	0.1318 ± 0.0177^*∗∗*^	0.1277 ± 0.0127^*∗*^

The values are presented as mean ± S.D. Significant differences with the NC group were designated as ^Δ^*P* < 0.05, ^ΔΔ^*P* < 0.01, with the M group ^*∗*^*P* < 0.05, ^*∗∗*^*P* < 0.01, with the WPP group ^#^*P* < 0.05, ^##^*P* < 0.01.

**Table 5:** Renal function indexes (mg/dL, *n* = 8).

	CRE	BUN	UA
NC	29.18 ± 3.25^*∗*^	6.76 ± 0.41^*∗∗*^	116.00 ± 18.49^*∗∗*^
M	31.98 ± 1.46^Δ^	9.38 ± 0.70^ΔΔ^	171.24 ± 43.83^ΔΔ^
JK	26.45 ± 4.43^*∗∗*^	7.75 ± 0.46^*∗∗*^	146.45 ± 29.43^*∗*^
SPP	26.78 ± 2.44^*∗∗#*^	8.35 ± 1.09^*∗∗*^	129.76 ± 32.90^*∗∗*^
WPP	28.39 ± 3.89^*∗∗*^	7.36 ± 0.48^*∗∗*^	167.16 ± 60.61^*∗*^

The values are presented as mean ± S.D. Significant differences with the NC group were designated as ^Δ^*P* < 0.05, ^ΔΔ^*P* < 0.01, with the M group ^*∗*^*P* < 0.05, ^*∗∗*^*P* < 0.01, with the WPP group ^#^*P* < 0.05.

**Table 6:** Sex hormone levels in each group (*n* = 8).

	T (ng/mL)	E_2_ (pg/mL)	FSH (m·IU/mL)	LH (m·IU/mL)
NC	12.13 ± 7.74	77.53 ± 17.70	1.41 ± 0.40	22.77 ± 15.19
M	0.76 ± 0.18^ΔΔ^	158.61 ± 71.22^ΔΔ^	4.69 ± 1.58^ΔΔ^	1.73 ± 0.41^ΔΔ^
JK	7.63 ± 10.94^*∗∗*^	96.79 ± 17.74^*∗*^	2.07 ± 1.02^*∗∗*^	3.09 ± 1.51^*∗*^
SPP	1.89 ± 1.45^*∗#*^	55.75 ± 14.10^*∗∗#*^	1.30 ± 0.75^*∗∗*^	5.92 ± 4.26^*∗*^
WPP	5.18 ± 3.62^*∗∗*^	42.72 ± 5.53^*∗∗*^	1.32 ± 0.74^*∗∗*^	9.58 ± 5.38^*∗∗*^

The values are presented as mean ± S.D. Significant differences with the NC group were designated as ^ΔΔ^*P* < 0.01, with the M group ^*∗*^*P* < 0.05, ^*∗∗*^*P* < 0.01, with WPP group ^#^*P* < 0.05.

**Table 7:** Biochemical indexes of seminal plasma in rats (*n* = 8).

	Fructose (mg/L)	*α*-Glucosidase (ng/L)	ACP (ng/L)	Zn (*μ*mol/L)
NC	4.16 ± 0.32	217.67 ± 12.86	4.48 ± 0.29	11.07 ± 0.58
M	3.70 ± 0.36^Δ^	169.56 ± 38.35^ΔΔ^	4.00 ± 0.22^ΔΔ^	10.44 ± 0.22^ΔΔ^
JK	4.34 ± 0.14^*∗∗*^	217.20 ± 11.48^*∗∗*^	5.53 ± 0.52^*∗∗*^	12.56 ± 1.33^*∗∗*^
SPP	4.46 ± 0.55^*∗∗*^	218.11 ± 9.49^*∗∗*^	5.15 ± 0.72^*∗∗##*^	11.32 ± 0.91^*∗∗*^
WPP	4.05 ± 0.28^*∗*^	225.19 ± 21.88^*∗∗*^	4.25 ± 0.10^*∗∗*^	10.82 ± 0.72

The values are presented as mean ± S.D. Significant differences with the NC group were designated as ^Δ^*P* < 0.05, ^ΔΔ^*P* < 0.01, with the M group ^*∗*^*P* < 0.05, ^*∗∗*^*P* < 0.01, with the WPP group ^#^*P* < 0.05, ^##^*P* < 0.01.

## Data Availability

The data used to support the findings of this study are available from the corresponding author upon request.

## Abbreviations

3-CQA: 3-Caffeoylquinic acid
4-CQA: 4-Caffeoylquinic acid
3,4-DiCQA: 3,4-Dicaffeoylquinic acid
5-FQA: 5-O-Feruloylquinic acid
RSD: Relative standard deviation
CRE: Creatinine
E_2_: Estradiol
FSH: Follicle-stimulating hormone
LH: Luteinizing hormone
3,5-DiCQA: 3,5-Dicaffeoylquinic acid
4,5-DiCQA: 4,5-Dicaffeoylquinic acid
CA: Caffeic acid
MRM: Multiple reaction monitoring
BUN: Blood urea nitrogen
ESI: Electron spray ionization
T: Testosterone
UA: Uric acid.
